# Surveillance of SARS-CoV-2 in Healthcare Workers Before and After COVID-19 Vaccination: A Cohort Study in a Primary Care Unit of Brazil

**DOI:** 10.3390/healthcare12222298

**Published:** 2024-11-17

**Authors:** Ana Cláudia Pinheiro Torres, Raissa Nogueira de Brito, Wildo Navegantes de Araújo, Priscilla Pedrette, Daiani Cristina Cilião Alves, Ana Izabel Passarella Teixeira, Carolina Carvalho Gontijo, Gustavo Adolfo Sierra Romero, Rodrigo Gurgel-Gonçalves, Walter Massa Ramalho

**Affiliations:** 1 Center for Tropical Medicine, University of Brasília, Brasília 70910-900, DF, Brazil; anacpinheirot@gmail.com (A.C.P.T.); wildo74@gmail.com (W.N.d.A.); romgustavo@gmail.com (G.A.S.R.); gurgelrg@hotmail.com (R.G.-G.); 2 Department of Anthropology, University of Georgia, Athens, GA 30602, USA; raissanogueirabrito@gmail.com; 3 Ceilândia Campus, University of Brasília, Brasília 70910-900, DF, Brazil; 4 National Institute for Science and Technology for Health Technology Assessment, Porto Alegre 90035-003, RS, Brazil; 5 Laboratory of Geography, Environment and Health, University of Brasília, Brasília 70910-900, DF, Brazil; priscillapedrette@gmail.com; 6 Laboratory of Molecular Diagnostics of University Hospital—EBSERH, University of Brasília, Brasília 70910-900, DF, Brazil; daianicristina@yahoo.com.br; 7 Campus Paranaíba, Federal University of Mato Grosso do Sul, Paranaíba 79070-900, MS, Brazil; anaipassarella@gmail.com; 8 Laboratory of Human Genetics, University of Brasília, Brasília 70910-900, DF, Brazil; carolinacarvalhogontijo@gmail.com

**Keywords:** monitoring, seroprevalence, SARS-CoV-2, COVID-19

## Abstract

Introduction: Healthcare workers (HCWs) are at higher risk of SARS-CoV-2 infection. Viral surveillance for early detection of COVID-19 is a critical strategy to understand this population’s infection dynamics and prevent transmission. The study examines SARS-CoV-2 infection and reinfection among HCWs vaccinated against COVID-19 working at a primary healthcare unit serving a disenfranchised community in Brazil. Methods: The study was conducted in Cidade Estrutural, Federal District, Brazil, between February and October 2021. Participants were interviewed and provided samples. A prospective open cohort study was used to analyze the frequency of SARS-CoV-2 infection and reinfection, and the vaccine-induced seroconversion. Nasopharyngeal swab specimen was collected from workers presenting with flu-like symptoms and subjected to RT-qPCR. Peripheral blood samples were also collected every 30 ± 2 days for eight months, starting from the day participants received their first dose of COVID-19 vaccine, and submitted to serological testing (IgM and IgG chemiluminescence). The frequencies of infection and reinfection (RT-qPCR positive results 90 days after the infection) were calculated along with their respective confidence intervals (95% CI). Results: Of the 128 workers, 61 (47.65%; CI: 39.19–56.25) reported probable SARS-CoV-2 infection before vaccination and 50 (39.06%; CI: 31.04–47.71) had SARS-CoV-2 infection after vaccination, confirmed by molecular test. Reinfection was identified in seven workers (7/50, 14%; CI: 6.95–26.18) based on the 90-day interval between results. The serological data from the 128 workers during the cohort indicated that 68 (53.12%; CI: 44.5–61.5) had IgG antibodies and 46 had IgM antibodies (35.93%; CI: 28.14–44.54) against SARS-CoV-2. SARS-CoV-2 infection was common in 56% of the community health workers (CHWs), 50% of registered nurses, and licensed vocational nurses (33%). Following the COVID-19 vaccination, the percentage of infections among HCWs decreased from 47.83% to 4.35%. Conclusion: These results demonstrate that (i) approximately 40% of the workers were infected with SARS-CoV-2 in 2021 and (ii) reinfections confirmed by RT-qPCR occurred in 14% of the HCWs after vaccination. The results provide valuable insights into the circulation of SARS-CoV-2 among HCWs in a primary care unit serving a minoritized community.

## 1. Introduction

The COVID-19 pandemic caused by SARS-CoV-2 has led to more than 700 million cases and 7 million deaths worldwide. Despite the pandemic being declared over, new cases of COVID-19 continue to be reported [1]. Healthcare workers (HCWs) working in primary care are on the front lines of the COVID-19 response and still face a high risk of infection due to frequent and close interactions with infected patients. Whether during the application of medical care, collection of biological samples, or administration of medications, they are highly exposed to the generation of aerosols, which is the main form of transmission of SARS-CoV-2, as well as exposed to all sorts of biological fluids that may contain viral particles [2]. During waves of COVID-19, positivity rates among HCWs were high, reported to be around 42.37% throughout the follow-up of the study of Buonafine [3]. However, in Caixeta’s study [4], the proportion of positivity in HCWs in samples collected in 2020 was much lower, at 21.6%.

The safety of these workers is crucial for their well-being and preventing transmission to uninfected patients and avoiding healthcare system backlog caused by absenteeism [5]. The surveillance of SARS-CoV-2 among HCWs is essential for monitoring their health, understanding the transmission dynamics, and evaluating their importance as a sentinel group for detecting virus variant shifts. Efficient monitoring of SARS-CoV-2 infections can help prevent high rates of COVID-19 and is crucial for identifying vulnerable groups and reducing the virus’s spread within healthcare facilities [6]. Despite COVID-19 vaccination having a robust protective effect against reinfection, the risk of SARS-CoV-2 infection persists among HCWs because of the occupational hazard. Reinfections can occur in fully vaccinated workers but with reduced severity and mortality [7,8].

Several factors have been identified as potential risk factors for SARS-CoV-2 transmission among HCWs, including job role, work environment, use of personal protective equipment, vaccination status, and concurrent community and household exposure. Studies have shown substantial variability in the prevalence of and risk factors for SARS-CoV-2 infection among HCWs, attributed to different job roles, exposure to COVID-19 patients, and healthcare settings [9]. However, the risk of SARS-CoV-2 exposure for HCWs is higher in the community than at their workplace [10]. HCWs in the Global South may face limited resources and workforce shortages, leading to overworked staff and reduced quality of care. These challenges underscore the need for targeted support and resources to protect HCWs in the Global South to ensure they can provide the highest quality of care during the pandemic. Monitoring infection and seroconversion among HCWs is crucial in identifying at-risk individuals, assessing the effectiveness of protective measures, and implementing timely interventions to mitigate the spread of SARS-CoV-2 [8,9,10].

This study investigates SARS-CoV-2 infection and reinfection and the vaccine-induced seroconversion in HCWs from a primary healthcare unit serving a disenfranchised community in Cidade Estrutural (RA XXV SCIA/Estrutural, DF, Brazil). This city, on the outskirts of the capital of Brazil, Brasília, was home to Latin America’s largest untreated refuse disposal site until its decommissioning in 2018 [11]. Our work describes the sociodemographic profile of the HCWs and evaluates their frequency of infection, cases of reinfection, and post-infection and -vaccination IgM and IgG detection in a prospective descriptive open cohort. The surveillance of SARS-CoV-2 in the healthcare workforce is a strategic approach for filling information gaps, understanding the virus’s behavior, and enabling early responses for the population.

## 2. Material and Methods

### 2.1. Study Design, Settings, and Ethical Considerations

**Figure 1:**
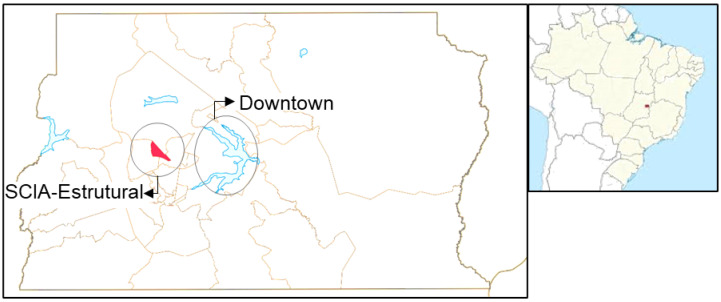
Study area: location of the primary healthcare unit in the Administrative Region of Cidade Estrutural (red) in the Federal District, Brazil. Downtown Brasília: the capital city of Brazil n: Source: https://pt.wikipedia.org/wiki/Distrito_Federal_%28Brasil%29, accessed on 15 August 2024.

This research was a prospective analytical cohort study conducted between February and October 2021 with the staff of a primary health care unit (HU) at Cidade Estrutural (RA XXV SCIA/Estrutural, DF, Brazil) (Figure 1). The Cidade Estrutural was home to the world’s second-largest untreated refuse disposal site for decades, which was closed in 2018. The dump was closed to help minimize outcomes related to improper waste disposal and to improve recycling techniques. However, even after its closing, the city continues to face numerous health issues such as dengue fever and waterborne diseases [12]. Currently, Cidade Estrutural is characterized by considerable social challenges, where many residents face economic difficulties, inadequate housing conditions, and limited access to essential sanitation services [11].

The committees approved this research in line with the Ethics in Research of the Faculdade de Medicina of Universidade de Brasília (CEP-FM/UnB, CAEE 39866620.4.0000.5558) and of Fundação de Ensino e Pesquisa em Ciências da Saúde (FEPECS/SES/DF, CAAE 40557020.6.3001.5553). All HCWs were invited to participate and signed an informed consent to participate in this study. This work was conducted according to the Ethical Principles for Medical Research in Human Subjects (Declaration of Helsinki) and Brazilian regulations (Resolution 466/12 Conep/CNS/MS).

### 2.2. Selection of Participants

**Figure 2:**
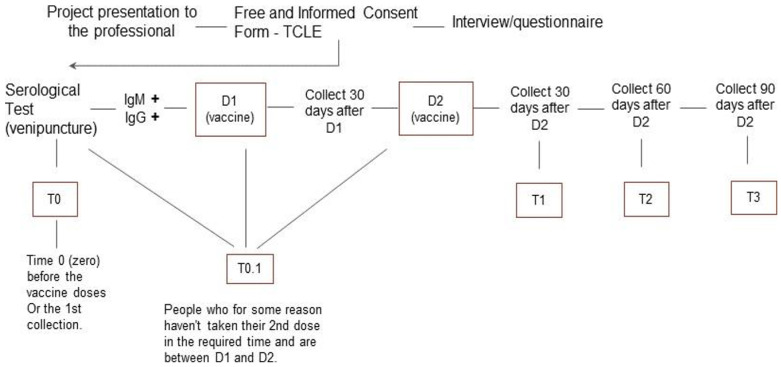
Flowchart depicting the sample collection scheme. Beginning on the day the participants received their first dose of the vaccine (D1), samples were collected, and interviews were conducted every 30 ± 2 days for eight months. If the participant had previously received a shot, D2 was considered the starting point. HWCs presenting with flu-like symptoms were tested for COVID-19 by RTq-PCR.

The target population was the team of 134 HCWs at the HU in Cidade Estrutural. The health care team is responsible for providing primary care services for the population of Cidade Estrutural, including vaccinations, examinations, outpatient clinical care, and scheduled appointments, among other services. All HCWs were invited to participate. The study included HCWs who worked in the HU during the period studied that voluntarily signed the informed consent, agreed to provide biological samples (nasopharyngeal swab specimen and peripheral blood), and answered a standardized questionnaire during the investigation. Participants were classified into three categorical groups: HCWs who (i) had not yet received the first dose of the COVID-19 vaccine (Coronavac or AstraZeneca) for any reason; (ii) had received the first dose of any COVID-19 vaccine; or (iii) had received two doses of any COVID-19 vaccine (during this initial period of the study, only Coronavac or AstraZeneca was available to HCWs [13]). In all three cases, the biological material and primary data were collected immediately preceding the scheduled vaccination date. Participants were interviewed to obtain sociodemographic characteristics and had a venous blood sample collected every 30 ± 2 days from the initial sample collection (Figure 2). Data on adverse effects were recorded, and serological analysis was performed at the same intervals. The follow-up period lasted eight months, during which any workers reporting flu-like symptoms were evaluated for COVID-19 using RT-qPCR at any time.

### 2.3. Serological Analysis and SARS-CoV-2 Detection

A total of 240 venous blood samples (approximately 4 mL each) were collected over a period of eight months. The samples were centrifuged at 5000 rpm (rotations per minute), and the serum was separated and stored at −80 °C until analysis. The qualitative detection of IgM and IgG antibodies against SARS-CoV-2 was performed using chemiluminescence microparticle immunoassay technology. The Abbott Architect Plus i2000SR was used to conduct tests detecting IgM against SARS-CoV-2 spike proteins (S) and IgG against SARS-CoV-2 nucleocapsid proteins (N). These assays were automated analyses performed by the Abbott machine. The antibodies against SARS-CoV-2 bind to antigen-coated microparticles, and then an acridinium-labeled anti-human antibody conjugated is added to create a new mix. The results are a chemiluminescent reaction measured in relative light units. If the test result was <1.4, the sample was considered negative; if it was 1.4, the test result was considered positive [14]. All tests were performed by the manufacturer’s instructions, including calibration and daily analysis of positive/negative controls, to meet the required quality criteria.

Molecular diagnosis was conducted on demand at the Laboratory Facilities of the Hospital Universitário de Brasília (HUB) to assess infection and reinfection using the nasopharyngeal swab samples collected. RNA was isolated using the EXTRACTA 32 kit (MVXA-P016 FAST) (Loccus, São Paulo, Brazil) in a Loccus automated extractor following the manufacturer’s instructions. SARS-CoV-2 was detected by the amplification of the genes E, RdRP, and N, as well as an internal control gene, according to the manufacturer’s protocol by the RT-qPCR Allplex™ 2019-nCoV Assay (Seegene Inc., Contagem, Brazil). RT-qPCR results were considered positive (SARS-CoV-2 RNA detected) when the internal control and at least two genes were amplified, negative (SARS-CoV-2 RNA not detected) when the internal control and none or only one gene was amplified, and inconclusive when the internal control did not amplify. Inconclusive RT-qPCR reactions were repeated once. Therefore, infection and reinfection criteria were based on RT-qPCR positive results.

### 2.4. Data Analysis

We assessed the frequency of infection and reinfection (positive results 90 days after an infection) among HCWs according to their sociodemographic characteristics. The study analyzed the frequency of SARS-CoV-2 at nine time points, both before and after vaccination, to assess infection and reinfection. We used the Hmisc package in R 4.2.1 software and the RStudio 2023.03.1.446 [15] interface.

## 3. Results

### 3.1. Characterization of the Population

From the 134 HCWs employed at the HU, 128 participated in the study. Four HCWs declined to participate in the study. Three HCWschose not to receive the vaccine but agreed to be monitored. During the study period, seven workers withdrew, and twelve new workers joined the study. A total of 128 workers were sampled at various stages of the study. A total of 27 were sampled before receiving the first dose of the vaccine, 109 before the second dose, 19 immediately after the second dose, and 99 on the 30th day (±2) after the second dose. Four HCWs received the AstraZeneca vaccine, while the remaining workers (124) were vaccinated with CoronaVac. Most workers had completed college education, earned more than six-figure minimum wages, and worked as nursing technicians or CHWs (Table 1).

### 3.2. SARS-CoV-2 Infections and Reinfections

Of the 128 workers, 61 self-reported probable SARS-CoV-2 infection (47.65%; CI: 39.19–56.25) before vaccination. A total of 50 (39.06%; CI: 31.04–47.71) HCWs had SARS-CoV-2 infection after vaccination, confirmed by RT-qPCR. Reinfection was identified in seven HCWs (14.00%; CI: 6.95–26.18), based on a positive RT-qPCR result after 90 days of a previous positive RT-qPCR result. The serological data from 128 HCW indicated that 68 had IgG antibodies (53.12%; CI: 44.5–61.5) and 46 had IgM antibodies (35.93%; CI: 28.14–44.54) against SARS-CoV-2 proteins.

Infections occurred in all ages, races, and educational groups (Table 1). No differences in infection were observed among the different income groups (Table 1). However, infections were identified in HCWs from other professions, mostly in community health workers (CHWs, 56%), registered nurses (50%), and licensed vocational nurses (33%) (Table 1). Following the vaccination, the percentage of infections among CHWs decreased from 47.83% to 4.35%.

Table 2 presents the number of HCWs with IgG and IgM positive based on the measurement time for both vaccinated and unvaccinated individuals. At the beginning of the study (T0), only 28 (22.05%) individuals had a positive IgG result before vaccination. At T1, 42 individuals who received the first dose of the vaccine tested positive for IgG (40.38%). The frequency of positive IgG gradually decreased in subsequent measurements (Table 2). A comparable pattern was observed for IgM, although to a lesser extent.

## 4. Discussion

The study found that 50 (~40%) HCWs in the HU located in Cidade Estrutural were infected with SARS-CoV-2 in 2021. CHWs were the most infected group. The study also showed a low frequency of reinfection among these workers after vaccination. These results contribute to our understanding of SARS-CoV-2 infection dynamics among HCWs of a HU serving a disenfranchised community in Brazil during the COVID-19 pandemic. The study of HCW infection is fundamental to understanding transmission dynamics and suggesting prevention strategies. The WHO [1] recommended that testing and isolating asymptomatic HCWs infected with SARS-CoV-2 from health care settings were critical for controlling COVID-19 transmission. This also directly impacts the quality of care and the mental health of HCWs, minimizing additional stress [4]. We found that approximately 40% of HCWs were infected with SARS-CoV-2 in 2021. Various studies have reported that prior to the first dose of COVID-19 vaccination, SARS-CoV-2 infections varied between 7 and 58% among HCWs, depending on the diagnostic method used [3,4,16]. It is worth noting that studies have demonstrated the effectiveness of combining serological methods with RT-qPCR to detect SARS-CoV-2, resulting in a more accurate diagnosis. In some suspected COVID-19 cases, when presenting with flu-like symptoms and having had close contact with confirmed cases, patients have been evaluated negative twice by RT-qPCR but positive for SARS-CoV-2-specific IgM and IgG antibodies. These findings suggest a promising strategy for preventing or controlling future cases [17]. During the post-vaccination stage, SARS-CoV-2 infections were reduced among HCWs, varying between 0.5 and 9% [18,19,20,21]. These values are marginally lower than those shown in our study. Surveillance based on accurate diagnostic methods allows for the prevention and management of COVID-19 symptoms, which can help prevent serious outcomes for HCWs, including death [17].

We detected SARS-CoV-2 infections in workers from over 18 different healthcare roles, with the majority being CHWs (56%). Association between job role and SARS-CoV-2 infection was found among healthcare personnel [7], although the risk of SARS-CoV-2 exposure for HCWs was more likely to have occurred in the community and/or their households rather than at their workplace [8]. The higher occurrence of SARS-CoV-2 infections in CHWs may be linked to their direct contact with the community and visits to households, where transmission occurs. Notably, a seroprevalence of 24% was observed in some regions of the Federal District of Brazil during the pandemic [22].

Our results show the dynamics of seroconversion in vaccinated individuals, indicating a positive immune response to the vaccine over time, with an initial increase at T1 and effective maintenance through T7. This analysis highlights the effectiveness of the vaccines in producing a detectable immune response in HCWs, as observed in other studies [21,22,23,24]. After vaccination, participants who had a previous, self-reported SARS-CoV-2 infection had higher antibody levels than those who did not self-report an earlier infection. This correlation between post-vaccination antibody levels and previous infection has been observed in other studies [24]. The effectiveness of the COVID-19 vaccine in HCWs was high, even against the Omicron variant (e.g., [25]). A reduction in COVID-19 cases in HCWs was observed in many studies [21,22,23,24]. For example, a significant decrease in new cases of COVID-19 among HCWs seven weeks after vaccination was observed, demonstrating the effectiveness of the vaccines [25,26].

COVID-19 infection among HCWs is a significant public health challenge that requires implementing more effective protective measures. A system where hospital-based occupational health services were adapted to offer a monitoring program with daily evaluations and treatment options for HCWs with SARS-CoV-2 has shown noteworthy results. Of the 4814 professionals enrolled, only 2% were hospitalized, and there were 6 deaths. The tracked professionals had lower rates of comorbidities, hospitalization, and mortality, indicating that this surveillance approach may be feasible [27].

The study was conducted during a government-declared state of emergency, which, in conjunction with a global shortage of supplies, caused delays in the arrival of diagnostic kits. As a result, there were frequent delays in the return of results, leading some participants to seek diagnostics in the private sector. Another limitation is related to the kit used during diagnosis, as we used a qualitative method available at the beginning of the cohort. It was not possible to measure the level of antibodies after exposure. Overcoming these challenges is critical to ensure the effectiveness of the vaccines and its effective contribution to pandemic control and the formulation of more effective and comprehensive health strategies in the future.

Surveillance offers the prospect of monitoring and follow-up, providing timely and effective health responses to health systems, populations, and governments. Incorporating routine surveillance as a public health policy could prevent future barriers to conducting research projects. COVID-19 surveillance provides an epidemiologic perspective for controlling virus transmission. Public health surveillance by community health workers is critical, especially in disenfranchised settings. HCWs play essential roles, including contact tracing and patient visits, in controlling infectious diseases such as HIV/AIDS, malaria, tuberculosis, Ebola, and COVID-19. Despite the challenges HCWs face, such as unfavorable psychosocial conditions, insufficient training and resources, investment in their well-being at work and infrastructure can significantly improve their work and the quality of public health surveillance [28,29].

## 5. Conclusions

Our results demonstrate that (i) approximately 40% of the HCWs were infected with SARS-CoV-2 after vaccination, primarily CHWs, and (ii) reinfections occurred in 14% of the HCWs. The results provide valuable insights into the circulation of SARS-CoV-2 among HCWs in a primary care unit caring for an unserved community of the capital of Brazil during the COVID-19 pandemic. In addition, the study indicates that some HCWs were still infected even after complete immunization. This finding is consistent with previous studies that have also shown a decline in vaccine effectiveness over time, highlighting the need for revaccination.

## Figures and Tables

**Table 1:** Demographic characteristics and SARS-CoV-2 infection data of the HCWs participating in the study according to age, race, education, income, and function.

Variables	Frequency	Percent	Infection (%)		
	*n*	%	Self-Reported	Infection	Reinfection
Age					
<24	5	3.9	0.0	20.0	0.0
25–34	15	11.7	46.7	46.7	13.0
35–44	48	37.5	52.1	47.9	8.0
45–54	46	35.9	52.2	34.8	7.0
55–64	14	10.9	35.7	21.4	0.0
Race					
Yellow	4	3.1	75.0	25.0	0.0
White	35	27.3	42.9	40.0	6.0
Mixed	72	56.3	50.0	40.3	7.0
Black	17	13.3	41.2	35.3	12.0
Education					
Elementary school	4	3.1	100	0.0	0.0
High school	24	18.7	75.0	80.0	20.0
Vocational education	4	3.1	50.0	50.0	0.0
College	100	78.1	45.0	61.8	20.0
Master’s degree	6	4.6	66.0	80.0	20.0
PhD	1	0.8	0.0	0.0	0.0
Income					
Up to USD 600.00	19	14.9	47.3	55.6	0.0
Up to USD 1200.00	20	15.6	35.0	40.0	15.0
Up to USD 1800.00	26	20.3	34.6	34.6	8.0
Up to USD 2400.00	26	20.3	57.7	42.3	8.0
>USD 2400.00	34	26.6	55.9	35.3	6.0
No answer	3	2.4	80.0	0.0	0.0
Profession					
Administrative worker	3	2.3	66.7	33.3	0.0
Administrative Technician	9	7.0	22.2	11.1	0.0
Community health workers	23	18	47.8	56.5	13.0
Cleaning worker	3	2.3	66.7	0.0	0.0
Dentist	5	3.9	60.0	60.0	0.0
Laboratory Technician	1	0.8	0.0	100	0.0
Licensed vocational nurses	24	18.8	50.0	33.3	8.0
Manager	3	2.3	66.7	33.3	33.0
Oral health assistant	1	0.8	100	0.0	0.0
Oral Health Technician	5	3.9	40.0	80.0	0.0
Other	9	7.0	22.2	11.1	0.0
Nutritionist	1	0.8	0.0	100	0.0
Pharmacist	1	0.8	100	0.0	0.0
Physiotherapist	2	1.6	100	0.0	0.0
Psychologist	1	0.8	0.0	0.0	0.0
Physician	10	7.8	70.0	30.0	20.0
Registered nurse	18	14.1	50.0	50.0	0.0
Social worker	1	0.8	0.0	0.0	0.0
Speech therapist	1	0.8	100	100	0.0
Vigilant	7	5.5	28.6	28.6	0.0

(*n*) Total number of participants in the study. Self-reported: HCWs reported that they had COVID-19. Infection: positive results for RT-qPCR. Reinfection: positive result 90 days after an infection.

**Table 2:** Number of healthcare workers participating in the cohort that tested positive for IgG and IgM SARS-CoV-2 over time (T).

Time (T) *	IgG Positive	IgG Negative	IgM Positive	IgM Negative	*n*
T0	28	99	28	99	127
T0_1/	3	9	3	9	12
T1	42	62	26	80	101
T2	38	64	12	89	104
T3	25	68	7	86	93
T4	24	66	9	81	90
T5	16	65	9	71	81
T6	12	57	12	57	69
T7	10	49	9	50	59

(*n*) Total number of participants in the cohort. * Described in Figure 2.

## Data Availability

The data collected and analyzed for this study are available from the corresponding author upon reasonable request.

## Abbreviations

COVID-19	Coronavirus disease of 2019
CHWs	Community health workers
HUB	Brasília University Hospital
HCWs	Healthcare workers
HU	Primary health unit
IEQ	Enzyme immunoassay of chemiluminescence
95% CI	95% confidence interval
IgG	Immunoglobulin G
IgM	Immunoglobulin M
RA	Administrative Region
RT-PCR	Reverse transcriptase reaction followed by polymerase chain reaction.
SARS-CoV-2	Severe acute respiratory syndrome coronavirus 2
SCIA	Complementary Industry and Supply Sector
UnB	University of Brasília

## References

[1] WHO (2024). COVID-19 Dashboard. https://data.who.int/dashboards/covid19/cases?n=o.

[2] Wilson A.M., Sleeth D.K., Schaefer C., Jones R.M. (2022). Transmission of Respiratory Viral Diseases to Health Care Workers: COVID-19 as an Example. Annu. Rev. Public Health.

[3] Buonafine C.P., Paiatto B.N.M., Leal F.B., de Matos S.F., de Morais C.O., Guerra G.G., Martuchelli M.V.V., Oliveira D.B.L., Durigon E.L., Soares C.P. (2020). High prevalence of SARS-CoV-2 infection among symptomatic healthcare workers in a large university tertiary hospital in São Paulo, Brazil. BMC Infect Dis..

[4] Caixeta D.A., do Carmo M.A.V., da Fonseca F.G., Nogueira D.A., Coelho L.F.L., Malaquias L.C.C. (2023). Seroprevalence of SARS-CoV-2 in hospital workers in the southern region of Minas Gerais state in Brazil: An analysis of the pre-vaccine period. Braz. J. Microbiol..

[5] Nashwan A.J., Mathew R.G., Anil R., Allobaney N.F., Nair S.K., Mohamed A.S., Abujaber A.A., Balouchi A., Fradelos E.C. (2023). The safety, health, and well-being of healthcare workers during COVID-19: A scoping review. AIMS Public Health.

[6] Padilha D.A., Souza D.S.M., Kawagoe E.K., Filho V.B., Amorim A.N., Barazzetti F.H., Schörner M.A., Fernandes S.B., Coelho B.K., Rovaris D.B. (2023). Genomic Surveillance of SARS-CoV-2 in Healthcare Workers: A Critical Sentinel Group for Monitoring the SARS-CoV-2 Variant Shift. Viruses.

[7] Pizarro A.B., Persad E., Durao S., Nussbaumer-Streit B., Engela-Volker J.S., McElvenny D., Rhodes S., Stocking K., Fletcher T., Martinet C. (2022). Workplace interventions to reduce the risk of SARS-CoV-2 infection outside of healthcare settings. Cochrane Database Syst. Rev..

[8] Cegolon L., Magnano G., Negro C., Larese Filon F., ORCHESTRA Working Group (2023). SARS-CoV-2 Reinfections in Health-Care Workers, 1 March 2020-31 January 2023. Viruses.

[9] Kobayashi T., Trannel A., Heinemann J., Marra A.R., Etienne W., Abosi O.J., Holley S., Dains A., Jenn K.E., Meacham H. (2022). Association between job role and coronavirus disease 2019 (COVID-19) among healthcare personnel, Iowa, 2021. Antimicrob. Steward Healthc Epidemiol..

[10] Pouquet M., Decarreaux D., Di Domenico L., Sabbatini C.E., Prévot-Monsacre P., Fourié T., Villarroel P.M.S., Priet S., Blanché H., Sebaoun J.-M. (2024). SARS-CoV-2 infection prevalence and associated factors among primary healthcare workers in France after the third COVID-19 wave. Sci. Rep..

[11] Cruvinel V.R.N., Marques C.P., Cardoso V., Novaes M.R.C.G., Araújo W.N., Angulo-Tuesta A., Escalda P.M.F., Galato D., Brito P., da Silva E.N. (2019). Health conditions and occupational risks in a novel group: Waste pickers in the largest open garbage dump in Latin America. BMC Public Health.

[12] Cruvinel V.R.N., Zolnikov T.R., Bashash M., Marques C.P., Scott J.A. (2019). Waterborne diseases in waste pickers of Estrutural, Brazil, the second largest open-air dumpsite in world. Waste Manag..

[13] Brasil, Agência Nacional de Vigilância Sanitária Anvisa Uso Emergencial das Vacinas: Linha do Tempo na Anvisa: Conheça as Principais Etapas para Autorização Temporária de uso Emergencial das Vacinas. https://www.gov.br/anvisa/pt-br/assuntos/noticias-anvisa/2021/uso-emergencial-das-vacinas-linha-do-tempo-na-anvisa.

[14] Lijia S., Lihong S., Huabin W., Xiaoping X., Xiaodong L., Yixuan Z., Pin H., Yina X., Xiaoyun S., Junqi W. (2020). Serological chemiluminescence immunoassay for the diagnosis of SARS-CoV-2 infection. J. Clin. Lab Anal..

[15] RStudio Team (2022). RStudio: Integrated Development for R. RStudio, PBC.

[16] Teixeira Mendes E., Neto D.G.P.V., Ferreira G.M., Valença I.N., Lima M.P.J.S., de Freitas M.F.M.B., Donalisio M.R., Melo M.C., Lazari C., Goes J. (2023). Impact of COVID-19 RT-PCR testing of asymptomatic health care workers on absenteeism and hospital transmission during the pandemic. Am. J. Infect. Control.

[17] Pessa Valente E., Cruz Vaz da Costa Damásio L., Luz L.S., da Silva Pereira M.F., Lazzerini M. (2020). COVID-19 among health workers in Brazil: The silent wave. J. Glob. Health.

[18] Bueno-Hernández N., Carrillo-Ruíz J.D., Méndez-García L.A., Rizo-Téllez S.A., Viurcos-Sanabria R., Santoyo-Chávez A., Márquez-Franco R., Aguado-García A., Baltazar-López N., Tomita-Cruz Y. (2022). High Incidence Rate of SARS-CoV-2 Infection in Health Care Workers at a Dedicated COVID-19 Hospital: Experiences of the Pandemic from a Large Mexican Hospital. Healthcare.

[19] Alamri S.S., Alsaieedi A., Khouqeer Y., Afeef M., Alharbi S., Algaissi A., Alghanmi M., Altorki T., Zawawi A., Alfaleh M.A. (2023). The importance of combining serological testing with RT-PCR assays for efficient detection of COVID-19 and higher diagnostic accuracy. PeerJ.

[20] Amit S., Beni S.A., Biber A., Grinberg A., Leshem E., Regev-Yochay G. (2021). Postvaccination COVID-19 among Healthcare Workers, Israel. Emerg. Infect. Dis..

[21] Arriola C.S., Soto G., Westercamp M., Bollinger S., Espinoza A., Grogl M., Llanos-Cuentas A., Matos E., Romero C., Silva M. (2022). Effectiveness of Whole-Virus COVID-19 Vaccine among Healthcare Personnel, Lima, Peru. Emerg. Infect. Dis..

[22] Nogueira de Brito R., Passarella Teixeira A.I., Carvalho Gontijo C., Da Silva Faria R., Massa Ramalho W., Sierra Romero G.A., Castro M., Pessoa V., Araújo Torres L., Pereira Leite L. (2023). Seroprevalence of SARS-CoV-2 and Vaccination Coverage among Residents of a Lower-Middle-Class Population in the Federal District, Brazil. Vaccines.

[23] Toniasso S.C.C., Fernandes F.S., Joveleviths D., Filho F.F.D., Takahasi A.Y., Baldin C.P., Pereira R.M., da Silva L.P., Brum M.C.B. (2021). Reduction in COVID-19 prevalence in healthcare workers in a university hospital in southern Brazil after the start of vaccination. Int. J. Infect. Dis..

[24] Goodwin B., Zia H., Lo D.F. (2023). Assessment of early and post COVID-19 vaccination antibody response in healthcare workers: A critical review. Epidemiol. Infect..

[25] Fonseca M.H.G., de Souza T.D.F.G., de Carvalho Araújo F.M., de Andrade L.O.M. (2022). Dynamics of antibody response to CoronaVac vaccine. J. Med. Virol..

[26] Gaio V., Santos A.J., Amaral P., Faro Viana J., Antunes I., Pacheco V., Paiva A., Leite P.P., Gonçalves L.A., Araújo L. (2023). COVID-19 vaccine effectiveness among healthcare workers: A hospital-based cohort study. BMJ Open.

[27] Crosby J.C., Lee R.A., McGwin G., Heath S.L., Burkholder G.A., Gravett R.M., Overton E.T., Locks G., Fleece M.E., Franco R. (2024). A COVID-19 monitoring process for healthcare workers utilizing occupational health. Occup. Med. (Chic Ill).

[28] Alhassan J.A.K., Wills O. (2024). Public health surveillance through community health workers: A scoping review of evidence from 25 low-income and middle-income countries. BMJ Open.

[29] Sato T.d.O., de Faria B.S.F., Albuquerque B.B., Silva F.L.D., Rohwedder L.S., de Azevedo R.T., Gonçalves J.S., Vieira L.M.S.M.d.A., Triches M.I., de Sousa R.A. (2022). Poor Health Conditions among Brazilian Healthcare Workers: The Study Design and Baseline Characteristics of the HEROES Cohort. Healthcare.

